# Human epidermal keratinocytes and human dermal fibroblasts interactions seeded on gelatin hydrogel for future application in skin *in vitro* 3-dimensional model

**DOI:** 10.3389/fbioe.2023.1200618

**Published:** 2023-06-23

**Authors:** Safa Tahri, Manira Maarof, Syafira Masri, Rohaina Che Man, Hatem Masmoudi, Mh Busra Fauzi

**Affiliations:** ^1^ Centre for Tissue Engineering and Regenerative Medicine, Faculty of Medicine, Universiti Kebangsaan Malaysia, Kuala Lumpur, Malaysia; ^2^ Research Laboratory LR12SP18 “Autoimmunity, Cancer, and Immunogenetics”, University Hospital Habib Bourguiba, Sfax, Tunisia; ^3^ Pathology Department, Faculty of Medicine, Universiti Kebangsaan Malaysia, Kuala Lumpur, Malaysia

**Keywords:** gelatin hydrogel, 3D *in vitro* skin model, genipin, keratinocytes, fibroblasts, biomaterial

## Abstract

**Introduction:** Plenty of biomaterials have been studied for their application in skin tissue engineering. Currently, gelatin-hydrogel is used to support three-dimensional (3D) skin *in vitro* models. However, mimicking the human body conditions and properties remains a challenge and gelatin-hydrogels have low mechanical properties and undergo rapid degradation rendering them not suitable for 3D *in vitro* cell culture. Nevertheless, changing the concentration of hydrogels could overcome this issue. Thus, we aim to investigate the potential of gelatin hydrogel with different concentrations crosslinked with genipin to promote human epidermal keratinocytes and human dermal fibroblasts culture to develop a 3D-*in vitro* skin model replacing animal models.

**Methods:** Briefly, the composite gelatin hydrogels were fabricated using different concentrations as follows 3%, 5%, 8%, and 10% crosslinked with 0.1% genipin or non-crosslinked. Both physical and chemical properties were evaluated.

**Results and discussion:** The crosslinked scaffolds showed better properties, including porosity and hydrophilicity, and genipin was found to enhance the physical properties. Furthermore, no alteration was prominent in both formulations of CL_GEL 5% and CL_GEL8% after genipin modification. The biocompatibility assays showed that all groups promoted cell attachment, cell viability, and cell migration except for the CL_GEL10% group. The CL_GEL5% and CL_GEL8% groups were selected to develop a bi-layer 3D-*in vitro* skin model. The immunohistochemistry (IHC) and hematoxylin and eosin staining (H&E) were performed on day 7, 14, and 21 to evaluate the reepithelization of the skin constructs. However, despite satisfactory biocompatibility properties, neither of the selected formulations, CL_GEL 5% and CL_GEL 8%, proved adequate for creating a bi-layer 3D *in-vitro* skin model. While this study provides valuable insights into the potential of gelatin hydrogels, further research is needed to address the challenges associated with their use in developing 3D skin models for testing and biomedical applications.

## 1 Introduction

Tissue engineering is defined as a new branch of knowledge that combines technologies from different research areas including biology, chemistry, engineering, medicine, pharmacy, and material science (Bas et al., 2021). In addition to the potential to solve the current health issue of organ and tissue failure, this multidisciplinary field can provide advanced *in vitro* model systems. Indeed, the importance of enhancing the physiological relevance of *in vitro* systems and expanding their applications has increased exponentially to replace animal experiments as well as many other applications. Therefore, intensified efforts have been made toward systematic development and evaluation of relevant, reliable and more robust non-animal models.

The first attempts in alternative testing are based on the three Rs (3R principles, i.e., replacement, reduction, and refinement) (Russell and Burch, 1960). The 3Rs philosophy promotes the quest for 1) the replacement of animals with non-living models, 2) the reduction in the use of animals, and 3) the refinement of animal use practices. In this context, the Organization for Economic Cooperation and Development (OECD) allowed *in vitro* procedure that may be used for the hazard identification of irritant chemicals. It is based on reconstructed human epidermis (RhE), which in its overall design closely mimics the histological, morphological, biochemical, and physiological properties of the upper parts of the human skin (Organisation for Economic Co-operation and Development, 2020); (Organisation for Economic Co-operation and Development, 2015). This alternative approach has been thoroughly evaluated, validated and approved as a successful alternative for animal experimentation.

The three-dimensional (3D) *in vitro* models are an advanced approach to develop a full-thickness skin models that shows significant potential to evidently advance the engineering of skin replacements. Indeed, two examples of verified 3D models that are offered for sale commercially are the EpiDerm (MATTEK, 2020) skin model and the EPISKIN (LOREAL, 2020). Thus far, plenty of materials have been studied for their application in skin tissue engineering. Among these materials, a widely employed biomaterial in this field is gelatin, a protein obtained by partially hydrolyzing collagen, the main protein found in the skin, bones and white connective tissues of animals (Imeson, 1992). Besides the fact that gelatin has therapeutic properties for drug research or drug delivery, it also plays an important role in tissue engineering. Specifically, since it has the ability to create 3D porous structures in which cells can grow, this biomaterial can imitate *in vivo* microenvironment conditions. In fact, gelatin has been used to culture different cancer and stromal cells (Nii, 2021), (Ertekin et al., 2022), (Nii et al., 2020).

Currently, gelatin-hydrogel is used to support skin regeneration (Yehkung et al., 2011); (Nicodemus and Bryant, 2008); (Hoffman, 2012). Zhao et al. (2016) and co-workers revealed that the mechanical and degradation properties of a developed gelatin hydrogel can be modified by changing the hydrogel concentration. Furthermore, all concentrations of hydrogel showed excellent cell viability (>90%) with increases in cell adhesion and proliferation that is proportional to the gelatin hydrogel concentration (Zhao et al., 2015). Additionally, the hydrogel is found to support keratinocytes growth, differentiation, and stratification into a reconstructed multilayered epidermis with adequate barrier functions (Zhao et al., 2016). The properties of this hydrogel suggest that the keratinocytes/fibroblasts filled hydrogels can be used as epidermal substitutes, wound dressings, or substrates to construct various *in vitro* skin models. Epidermal keratinocytes and dermal fibroblasts that interact together can actively participate in cutaneous immune responses and are two of the major cell types that respond to the inflammatory phase in the cutaneous repair/regeneration process. Despite the promising results, it is still hard to mimic the human body conditions and properties and the gelatin-hydrogel suffers from poor mechanical properties and high degradation rate that maybe not suitable for 3D *in vitro* keratinocytes and fibroblasts culture and new 3D *in vitro* models need to be validated. To improve mechanical features of our gelatin hydrogels, we used genipin as a natural crosslinker. Genipin is a molecule extracted from the fruit of the gardenia plant that has been used to crosslink a variety of protein and polysaccharide matrices, including gelatin for drug-delivery applications (Liang et al., 2003); (Wei et al., 2007); (Dare et al., 2009); (Ko et al., 2009).

In short, gelatin may serve as an effective platform to support culture systems, which is a step towards the design of more accurate 3D *in vitro* skin models and it paves the way for investigating the performance of a wide range of chemical and pharmaceutical safety assessment in the future on in vivo-like and animal-free approaches. Hence, this study aims to characterize the physical-chemical parameters for gelatin hydrogel crosslinked with genipin and to evaluate the biocompatibility of the engineering 3D keratinocytes and fibroblasts culture seeded on bioscaffold prior to the formation of a matured bilayer co-culture for future application *in vitro* 3D skin model.

## 2 Materials and methods

### 2.1 Fabrication of gelatin hydrogel crosslinked with genipin

Gelatin hydrogel was fabricated from Nitta gelatin powder (Nitta Gelatin Inc^®^, Japan). The gelatin was mixed with distilled water with a magnetic stirrer until homogenized at 250 rpm at 40°C for 30 min to remove air bubbles and to completely blend after adding the genipin (FUJIFILM Wako Pure Chemical Corporation^®^, Japan). Then, 2 mL of the gelatin mixture was added to each well of a 12-well plate. The mixture was incubated at room temperature to initiate gelation to form the 3D constructs, which were termed gelatin10%-genipin0.1% (CL_GEL10%), gelatin8%-genipin0.1% (CL_GEL8%), gelatin5%-genipin0.1% (CL_GEL5%) and gelatin3%-genipin0.1% (CL_GEL3%) (*n* = 3, N = 3). Gelatin non crosslinked was used as control (NC_GEL10%), (NC_GEL8%), (NC_GEL5%), and (NC_GEL3%).

The gross morphology and microstructure of CL_GEL5% and CL_GEL8% constructs were observed via scanning electron microscopy (SEM; Quanta FEG 450, FEI; Eindhoven, North Brabant, Netherlands). The SEM analysis was performed to show the characteristics of the surface and porosity of different groups.

### 2.2 Physical and chemical characterization

#### 2.2.1 Porosity of gelatin hydrogel

The percentage of porosity of the gelatin hydrogel was measured by the below formula (School of Chemical Engineering, 2019). The measuring cylinder with the ethanol was recorded as V. The initial weight of the lyophilized gelatin hydrogel was recorded as *Wd*. The final weight of the immersed gelatin hydrogel in the 99.5% ethanol after 24 h was recorded as *Ws*.

$$Porosity\;\left(\%\right)=\frac{Ws-Wd}{\rho \;x\;V}X\;100\%$$

Where *ρ* = density of absolute ethanol.

#### 2.2.2 Swelling ratio

The formula below was used to calculate how much volume of water the gelatin hydrogel can absorb. The bio-scaffold was weighed in a dry condition (*Wd*). After dry weight, the bio-scaffold was immersed into the phosphate buffer saline (PBS 1X, pH 7.4) for 6 h. The wet bio-scaffold (*Ws*) was weighed and recorded at a constant time period (Maji et al., 2016).

$$Swelling\;ratio\left(S\right)=\frac{Ws-Wd}{Wd}X\;100\%$$

#### 2.2.3 Biodegradation

To observe the degradation of the gelatin hydrogel, the gelatin hydrogel was weighed first (*W*_0_) after pre-freezing at −80°C for 6 h and freeze-drying. The gelatin hydrogel was immersed in the diluted enzyme 0.0006% collagenase type I (Worthington^®^, NJ, United States) prepared in phosphate buffer saline (PBS 1X) and recorded in three different times 2, 4, and 24 h (t), which imitated the human body’s enzyme. After that, the gelatin hydrogel was washed using distilled water and pre-freeze at −80°C. The gelatin hydrogel underwent the freeze-drying process and its final weight was measured (*W*_t_). The biodegradation rate and the weight loss were calculated by using the following formulas:

$$Degradation\;rate\;\left(mg/h\right)=\left(W_{0}-W_{t}\right)/t$$

$$Weight\;Loss\;\left(\%\right)=\left[\left(W_{0}-W_{t}\right)/W_{0}\right]\times 100$$

#### 2.2.4 Degree of crosslinking

The ninhydrin assay (Sigma-Aldrich^®^, Saint Louis, MO, United States) was used to determine the degree of crosslinking of the sample. 10 mg of the sample and 200 µL of ninhydrin reagent were added into a clean test tube. After the vortex, the sample which was covered by the aluminum foil was boiled for 2 min. The sample was cooled before 200 µL of 95% ethanol was added into the test tube. We used the spectrophotometer reader (Bio-Tek^®^, Power Wave XS, Boston, United States) at 570 nm to measure the absorbance of the sample and we compared the control sample without crosslinking and the sample with crosslinking by using the ninhydrin assay.

#### 2.2.5 Contact angle

The gelatin hydrogel solutions were put on the glass slide and dried overnight. A water droplet was dropped on the glass slide in order to measure the angle of the gelatin hydrogel in contact with the surface of the glass slide by using ImageJ software (National Institute of Health, V1.5, Bethesda, MA, United States) to determine the surface wettability.

#### 2.2.6 Compression and resilience

The formulas below were used to calculate the compression ratio and the resilience ratio. The gelatin hydrogel was compressed using a tensile testing analyzer (Instron, Norwood, MA, United States) and then released in water for 5 min to observe the resilience. The hydrogel was compressed and released 3 times which needed to take a picture for every time.

$$Compression\;ratio\;\left(C\%\right)=\left(A_{i}\;–\;A_{c}\;/\;A_{i}\right)\;x\;100$$

$$Resilience\;ratio\;\left(R\%\right)=\left(A_{f}\;/\;A_{c}\right)\;x\;100$$

Where, A_i_: Area of thickness before compression. A_c_: Area of thickness after compression; A_f_: Area of thickness after resilience.

#### 2.2.7 Fourier transform infrared spectrometry

The chemical structure of the constructs was characterized using Fourier transform infrared spectrometry (FTIR) (IR Prestige-21, Shimadzu^®^, Kyoto, Japan) through functional group identification. The CL_GEL5% and CL_GEL8% constructs were prepared as described above, and the FTIR spectra of the constructs (*n* = 1) was recorded in the frequency range of 600–4,000 cm^−1^. The data were analyzed using Shimadzu IR Solution FTIR (spectroscopy) software (Shimadzu^®^).

#### 2.2.8 X-ray diffraction

The crystallographic structure analysis of the CL_GEL5% and CL_GEL8% biomaterials was performed by X-ray diffractometer (Bruker^®^, D8 Advance, United Kingdom) with diffraction angle (2θ) in the range of 0^◦^ to 80^◦^. The obtained diffractogram was evaluated by using the integrated software (Diffrac. Suite EVA, V4.0, Bruker^®^, Coventry, United Kingdom).

### 2.3 Biocompatibility characterization

#### 2.3.1 Cell isolation and culture (keratinocytes and fibroblasts)

The six patients underwent abdominal surgery. The redundant skin samples were collected at Hospital Canselor Tuanku Mukhriz (HCTM) (Ethics approval number: UKM 1.5.3.5/244/FF-2015-376). The sample collection based on the inclusion and exclusion criteria listed in Table 1.

**TABLE 1:** Inclusive and exclusive criteria.

Inclusive criteria	Exclusive criteria
• The patient needs to do the abdominal surgery	• Severe infections and/or ongoing antibiotic treatment
• Age is between 11 and 60 years old	
• The patient does not suffer from any chronic diseases	

After collecting the redundant skin samples from patients who underwent abdominal surgery, the unwanted materials such as hair, fat, and debris were cleaned from the 3 cm^2^ of skin samples. The samples were cut into a few small pieces with approximately 2 mm^2^. The cut pieces were incubated with 0.6% of collagenase type 1 (Worthington^®^, NJ, United States) in the 37°C for digestion for 2–4 h. The cells were dissociated or degraded by using 0.05% of Trypsin-EDTA (Gibco^®^, CA, United States) for 8–10 min. The human epidermal keratinocytes (HEKs) and human dermal fibroblasts (HDFs) contained in the digested skin were resuspended in the co-culture medium at a 1:1 ratio (a mixture of HEKs growth medium; Epilife^®^ (Gibco^®^, NY, United States) and HDF growth medium; F-12:Dulbecco’s Modified Eagle Medium (Gibco^®^, United States) supplemented with 10% fetal bovine serum (FBS; Biowest^®^, MO, United States) (FDC). In the six-well culture plate, we seeded the medium in three wells that the surface area is 9.6 cm^2^/well at 37°C in 5% CO_2_. By using the differential trypsinization, the fibroblasts were separated from the co-cultured keratinocytes after the waste medium reached 70%–80% confluence. The detached fibroblasts were re-cultured with FDC in a T75 flask. The keratinocytes were propagated in a six-well culture plate and the desired number of cells was obtained by the sub-culture fibroblasts and keratinocytes for further analysis. Three technical replicates were performed for each biological replicate.

#### 2.3.2 Cell attachment

HDFs (5 × 10^4^) and HEKs (15 × 10^4^) were seeded on hydrogels of different formulations, which were presoaked in F12: DMEM (Gibco^®^, NY, United States) and Epilife^®^ (Gibco^®^, NY, United States) with supplements, respectively, overnight. The cells were allowed to attach at 37°C with 5% CO_2_. The hydrogel was washed gently with Dulbecco’s Phosphate Buffer Saline (DPBS) (Sigma^®^, MO, United States) after 24 h. The remaining (unattached) cells in DPBS were counted using a hemocytometer and 0.4% trypan blue solution (Sigma^®^, MO, United States). The percentage of cell attachment was calculated as per the equation below:

Cell attachment (%) = [(Initial cell seeding − number of cells in DPBS)/Initial cell seeding] x 100.

#### 2.3.3 Cell toxicity assessment

Cytotoxicity test was performed towards HEKs and HDFs via LIVE/DEAD cytotoxicity assay for mammalian cells (Thermo Fisher Scientific^®^, MA, United States). The hydrogels were fabricated in a 48-well culture plate by using sterile gelatin and genipin solution. Immediately after polymerization, 5 × 10^4^ HDFs and 15 × 10^4^ HEKs at passage three (P3) were seeded on the top of hydrogel prior to the incubation for 24 h. Cell toxicity was examined by using a fluorescence microscope (Nikon^®^ A1R-A1, Japan) at ×10 magnification after treatment with 500 µL of a mixture of 2 mM acetomethoxy derivative of calcein (calcein-AM) and 4 mM ethidium homodimer-1 (EthD-1) at 37°C for 30 min.

#### 2.3.4 Viability and proliferation evaluation

The viability and proliferation of HEKs and HDFs were evaluated by using 3-(4,5-dimethylthiazol-2-yl)-2,5-diphenyltetrazolium bromide (MTT) (Thermo Fisher Scientific^®^, MA, United States). Briefly, 5 × 10^4^ HDFs and 15× 10^4^ HEKs at P3 (Nike et al., 2021); (Xi Loh et al., 2018) were seeded on the top of hydrogel according to the protocol previously described elsewhere (Masri et al., 2023); (Mh Busra et al., 2019) and MTT reagent was added after 1, 3, 5 and 7 days of incubation prior to the dimethyl sulfoxide (DMSO) addition as dissolution reagent. The absorbance was recorded by using a spectrophotometer reader (Bio-Tek^®^, Power Wave XS, Washington, United States) at 540 nm at specific time intervals. The cellular viability at day 1 was used as an indicator of the efficiency of HDFs and HEKs attachment. The total number of cells was calculated using the standard curve.

#### 2.3.5 Cell migration

The migration ability of the HDFs and HEKs in 3D hydrogels was evaluated by using gelatin-hydrogel models in which cells were seeded. The net migration of the cell population was measured from an upper chamber to a lower chamber through a microporous membrane. Subsequently, a 1 cm^2^ of each hydrogel was used for cell seeding in 48 well-plates. The cells were stained with blue cell tracker (Hoescht dye, Invitrogen^®^, MA, United States) and green cell tracker (Green dye, Invitrogen^®^, MA, United States) and incubated for 30 min in 37°C. HDFs and HEKs were stabilized for 2 and 24 h, respectively. Next, 5 × 10^4^ HDFs and 15× 10^4^ HEKs stained with green dye were seeded on the top of the scaffolds of each group and 5 × 10^4^ HDFs and 15× 10^4^ HEKs stained with blue dye were seeded in 48-well plate. The fluorescent dye retained in the cells allowing for multigenerational tracking of cellular movements. Meanwhile, the gelatin-cells constructs were monitored at day 1, 3, 5 and 7 to observe the cell migration from the transplanted site to the surrounding matrix. Cell migration images were captured using Nikon A1R confocal microscope (Nikon^®^, Japan), and cell migration distances from the hole edge to the cell outgoing front in all directions were measured via image analysis software (ImageJ) (National Institute of Health, V1.5, MA, United States). Afterward, the average migration distance was calculated for statistical analysis.

#### 2.3.6 Immunocytochemistry staining for proliferative cells

Keratinocytes and fibroblasts were fixed with 4% paraformaldehyde (Sigma-Aldrich^®^, MO, United States) for at least 15 min, permeabilized with 0.1% Triton X-100 solution (Sigma-Aldrich^®^, MO, United States) for 20 min, and blocked with 10% goat serum (Sigma-Aldrich^®^, MO, United States) for 1 h at 37°C. Next, the cells were incubated with mouse collagen type 1 monoclonal antibody (COL-I) (Abcam^®^, United Kingdom), rabbit alpha-smooth muscle actin antibody (α-SMA) (Abcam^®^, United Kingdom) and rabbit cytokeratin 14 antibody (CK-14) (Abcam^®^, United Kingdom) overnight at 4°C. On the next day, the cells were incubated with goat anti-mouse IgG Alexa Fluor 594 (Red-fluorescent dye, Invitrogen^®^, MO, United States) and goat anti-rabbit IgG Alexa Fluor 488 (Green-fluorescent dye, Invitrogen^®^, MO, United States) for 2 h at 37°C in dark. The cells then were counterstained with DAPI (Dako^®^, Denmark) for 20 min at room temperature and observed using Nikon A1R confocal microscope (Nikon^®^, Japan) for expression of collagen type 1, α-SMA and cytokeratin 14.

### 2.4 Develop a two-layer skin construct

#### 2.4.1 Preparation of the two-layer skin construct

The ability of the fibroblasts and keratinocytes to migrate into a gelatin hydrogel was compared in four-time intervals (on days 1, 3, 5 and 7 after cell seeding) and the CL_GEL5% and the CL_GEL8% gelatin hydrogels were selected for the preparation of the two-layer skin construct. Thus, a two-layer cell construct was composed of a CL_GEL5% and CL_GEL8% gelatin hydrogel pre-seeded with HDFs. A 1.9 cm^2^ of gelatin hydrogel samples were fitting into 12-well cell culture plates and seeded with HDFs. The fibroblasts were seeded on the scaffold at a density of 50,000 cells, (Nike et al., 2021); (Mh Busra et al., 2019) and were cultivated in FD supplemented with 10% of FBS and 1% of antibacterial-antimycotic (Gibco^®^, China). After 3 days of fibroblast cultivation, the collagen hydrogel was prepared on the fibroblast-seeded membrane, and the fibroblasts started to migrate into the hydrogel. The keratinocytes were seeded at a density of 150,000 cells after 4 days of gelatin hydrogel preparation (already seeded with of fibroblasts). After the keratinocytes had been seeded, the medium was replaced by FD and Epilife with supplements, as mentioned above. The medium was changed every 2 days. After 7, 14 and 21 days of fibroblast cultivation, the immunohistochemistry (IHC) and hematoxylin and eosin staining (H&E) were performed in order to evaluate the reepithelization of the skin constructs.

#### 2.4.2 Hydrogel frozen section technique

The frozen section method is a rapid and efficient technique for soft tissue analysis. Using a cryostat, the portion of hydrogels (1 cm diameter) were immediately frozen. The freezing procedure normally takes a few minutes and preserves the original condition of the tissue. Next, using a microtome, the hydrogels were sliced into thin sections with 4 μm thickness. Sectioned hydrogels were subjected for hematoxylin and eosin (H&E) and immunofluorescent (IF) staining to make them visible under microscope.

#### 2.4.3 Hematoxylin and eosin staining

The H&E staining was used to detect the nucleus and extracellular matrix (ECM) protein, allowing the identification of structural features of the epidermis and dermis layer. The H&E staining was carried out according to standard protocol. The hydrogel samples were first fixed in a solution such as 10% formalin, to preserve the hydrogels. First, the hydrogel samples were embedded in optimal cutting temperature (OCT) compound and snap-frozen in liquid nitrogen or dry ice. The frozen tissue block was then sectioned using a cryostat to a thickness of 4 μm and left for air dried. The hydrogel-sectioned slides were then dipped in H&E solution for 5–10 min. Next, the slides were then rinsed in distilled water and immersed in eosin solution for 30 s. Following that, the slides were rinsed with distilled water again before being dehydrated with graded alcohol solutions (70%, 90%, and 100% ethanol). Lastly, the slides were cleaned using xylene, and mounted with a coverslip using a mounting medium and ready to be observed under light microscope.

#### 2.4.4 Immunofluorescent staining

IHC was carried out using specific markers, cytokeratin 14 and collagen type 1 (Abcam^®^, MA, United States) to evaluate, respectively, the regenerated skin’s maturity for keratinocytes and the fibroblasts maturity. The hydrogels were then sectioned with a cryostat and mounted on glass slides. The fixed tissue sections were then blocked with 10% goat serum (Sigma Aldrich^®^, MO, United States) to prevent non-specific binding of the primary antibody. The tissue sections were incubated with a primary antibody that specifically binds to the protein of interest. The tissue sections are then incubated with a secondary antibody that recognizes the primary antibody Alexa 488 (Abcam^®^, MA, United States). The labelled secondary antibody is detected using fluorescence microscopy. Finally, the tissue sections counterstained with DAPI to visualize the nuclei and provide morphological context.

### 2.5 Statistical analysis

The quantitative results were shown as mean ± standard error mean (SEM). Statistical analyses were performed using GraphPad Prism 7.0 (GraphPad Software, United States) and the results were analyzed using one-way analysis of variance (ANOVA). The difference between groups is significant if *p* < 0.05.

## 3 Results

### 3.1 Characterization of hydrogels

#### 3.1.1 Gross appearance

**FIGURE 1:**
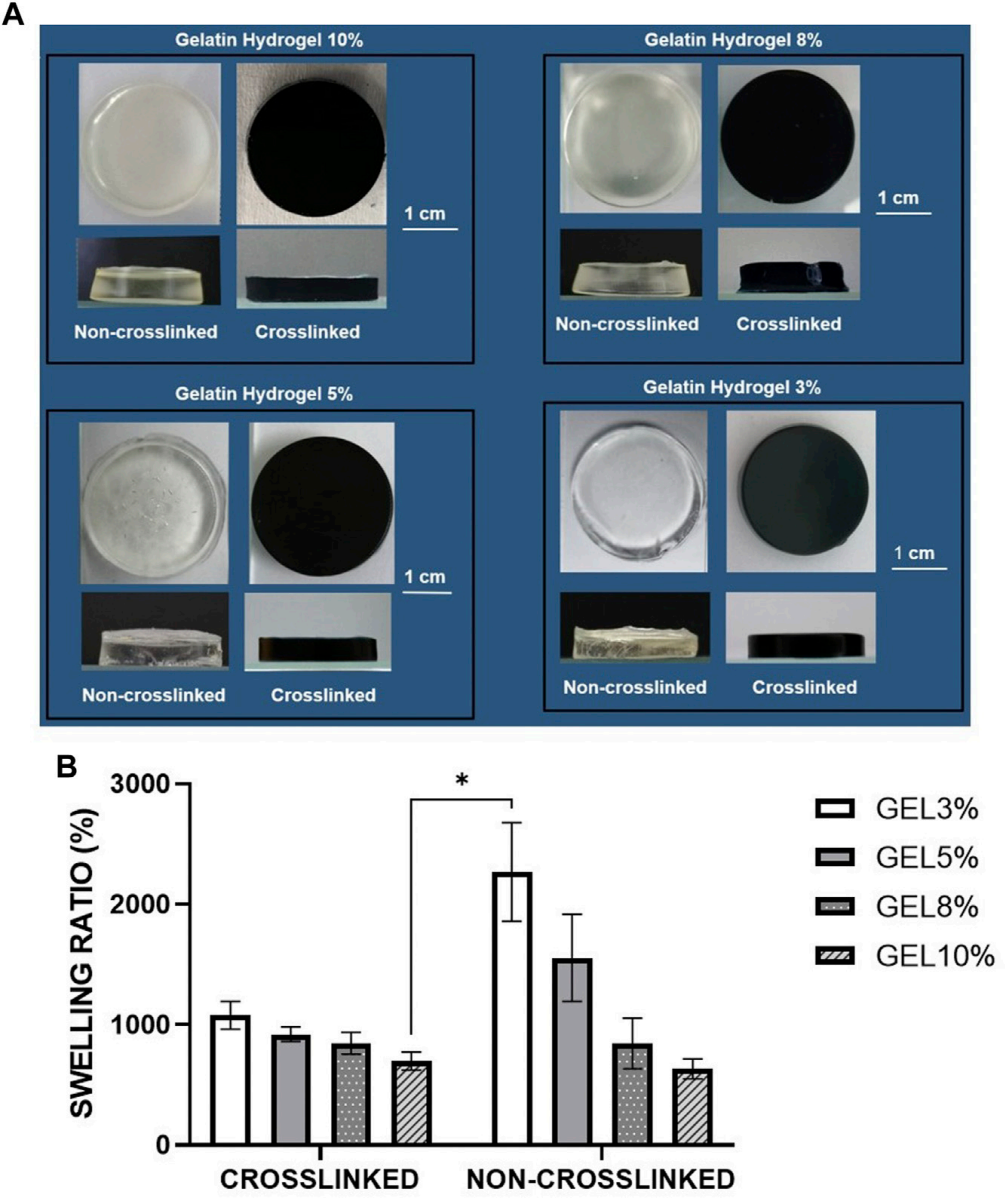
**(A)** Gross appearance of different gelatin hydrogel groups (3%,5%,8%, and 10%) non-crosslinked and crosslinked with 0.1% genipin at room temperature (22°–24°C). The crosslinked groups with 0.1% genipin showed greenish blue color while the non-crosslinked groups showed transparent structure. **(B)** Physical evaluation: swelling ratio of gelatin hydrogel scaffolds, i.e., GEL3%, GEL5%, GEL 8%, and GEL10% with and without genipin crosslinking. The non-crosslinked groups demonstrated higher swelling properties compared to crosslinked groups. *Represents the significant difference between crosslinked and non-crosslinked hydrogel (N = 3, *n* = 3).

The gelatin hydrogel scaffolds were fabricated with four different concentrations of gelatin crosslinked or non-crosslinked with genipin, respectively (CL_Gel 10%—CL_GEL 8% - CL_GEL 5% and CL_GEL 3%) and (NC_Gel 10% - NC_GEL 8% - NC_GEL 5% and NC_GEL 3%). Figure 1A shows the gross appearance of gelatin hydrogel with or without crosslinking. The scaffold crosslinked with genipin showed a greenish blue color as compared to the non-crosslinked one. In contrast, the non-crosslinked hydrogel showed a transparent structure. The crosslinked hydrogel was semi-solid gel and showed a smooth surface. The structure of the gel appeared to be more solid in the scaffolds with the highest concentration of gelatin. The same observation was observed in the non-crosslinked groups. Despite using the same material, these scaffolds vary in their gross appearance.

#### 3.1.2 Swelling ratio

The swelling ratio was determined as shown in Figure 1B. The NC_GEL 3% hydrogel scaffold demonstrated the best swelling property (2268.63 ± 136.9%) compared to the other experimental groups followed by the NC_GEL 5% (1556.05 ± 120.98%) and the CL_GEL 3% (1079.52 ± 38.42%). Meanwhile, the NC_GEL 10% hydrogel had the poorest swelling property (633.22 ± 27.4%), followed by CL_GEL 10% (698.52 ± 24.88%) and NC_GEL 8% (844.40 ± 70.52%). As the ratio of the polymers in the hydrogel was changed, keeping the crosslinker constant, swelling is increased. It was also found that the sample was unstable in the aqueous solution and was challenging to handle. It can be easily observed by the large error bars especially in the non-crosslinked groups.

One of the desired properties that plays a crucial role in cell adhesion and development as well as transfer of nutrients and metabolites via the hydrogel (Rodríguez-Vázquez et al., 2015), is water permeability. It was reported that the ability of the bioscaffold to absorb fluid 80 times over its initial weight is adequate for skin tissue engineering (Luangbudnark et al., 2012). The hydrogel concentration in our study caused a growing proportion of swelling in all constructions where the ability of CL_GEL8% to swell was almost 100 times over its initial weight, which is consistent with the gelatin’s microporous structure and porosity.

#### 3.1.3 *In vitro* biodegradation

**FIGURE 2:**
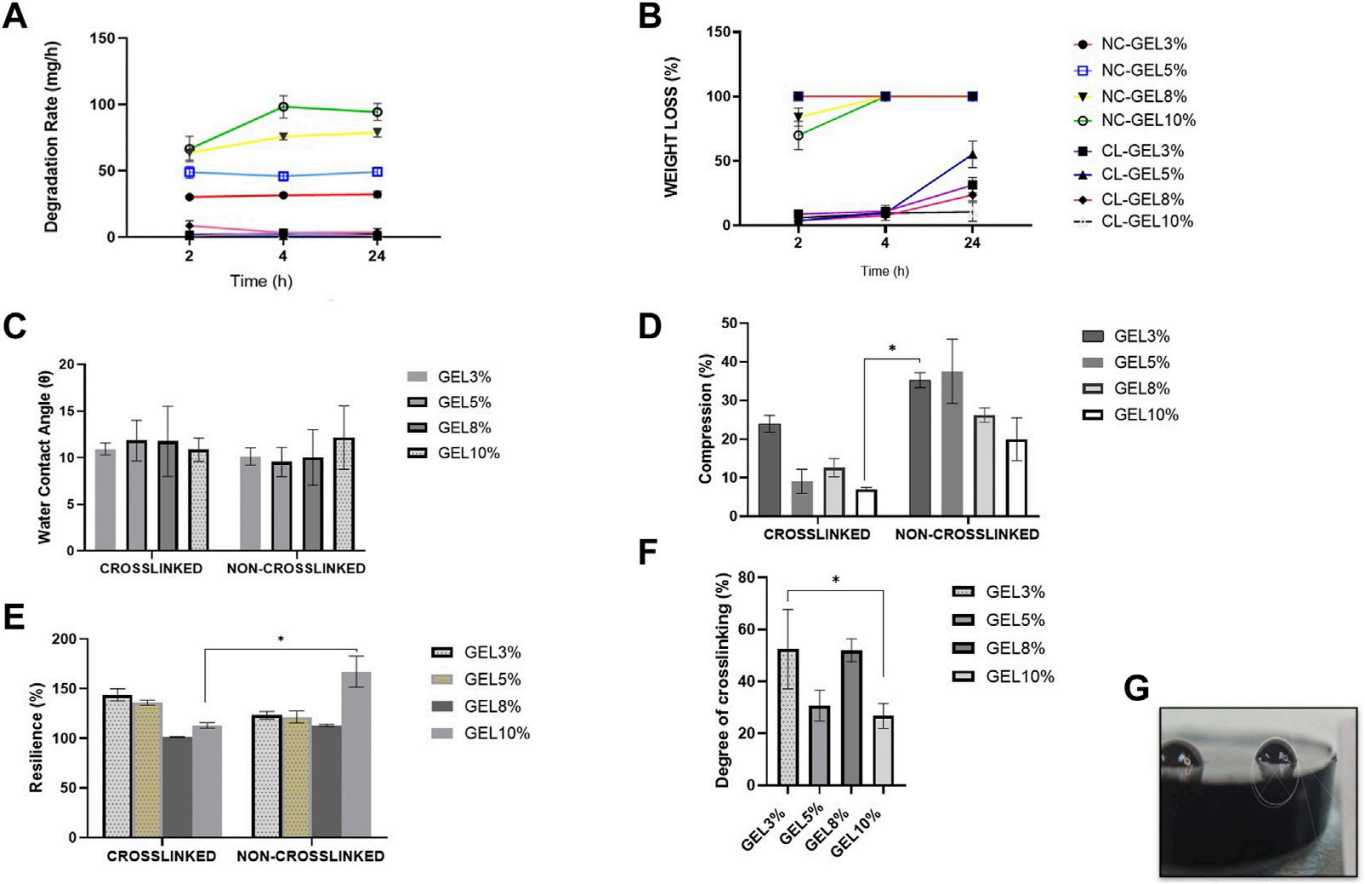
**(A)** Biodegradation rate and **(B)** weight loss at 37°C of the fabricated scaffolds compared to control at various times (2, 4, and 24 h). **(C)** Water contact angle of the fabricated scaffolds at room temperature. **(D)** compression and **(E)** resilience properties of the different hydrogel formulations compared to control. **(F)** Degree of crosslinking of the fabricated scaffolds. **(G)** measurement of water contact angle with ImageJ software * Represents the significant difference between groups.

Given that cells seeded in a hydrogel may secrete several proteases, such as collagenase, which could lead to hydrogel degradation, the degradation performance of gelatin hydrogel was evaluated. The degradation test was carried out in all groups. The biodegradation rates of the scaffolds were evaluated using the enzymatic degradation approach, as shown in Figure 2A. The non-crosslinked scaffolds (NC_GEL10%, NC_GEL8%, NC_GEL5%, and NC_GEL3%) together, were able to degrade faster (59.59 ± 1.45 mg/h) than crosslinked gelatin hydrogels (CL_GEL10%, CL_GEL8%, CL_GEL5%, and CL_GEL3%) together (2.58 ± 0.47 mg/h). The addition of genipin was able to decrease the degradation rate in the scaffolds dramatically. The weight loss test was carried out in all groups as shown in Figure 2B. After 2 h, the weight loss of the non-crosslinked groups was (69.85% ± 3.68%), and (83.97% ± 2.3%) in NC_GEL10% and NC_GEL8%, respectively and after 24 h, the weight loss of the non-crosslinked groups was 100%. After 2 h, the highest weight loss percentage in crosslinked group was in CL_GEL3% (8.87% ± 0.53%) and the lowest value was in CL_GEL8% (3.72% ± 0.17%). After 4 h, the CL_GEL3% registered the highest value of weight loss (10.94% ± 0.38%) and CL_GEL8% (7.61% ± 0.29%) registered the lowest value. And after 24 h, the CL_GEL5% showed the highest value (55.15% ± 3.41%) followed by CL_GEL3% (31.27% ± 1.96%) and CL_GEL8% (23.62% ± 1.52%) and CL_GEL10% (10.49% ± 2.39%). It seems that, the gelatin hydrogel concentration significantly helps in decreasing the weight loss. Comparing the crosslinked groups, the increase of gelatin hydrogel concentration helps in slowing down the weight loss process in crosslinked formulation.

Another way to improve the mechanical properties of the hydrogels is by using biodegradable synthetic scaffolds. The biodegradable hydrogel would serve as a substrate for the initial attachment and growth of fibroblasts and keratinocytes (MacNeil, 2007). Our results showed that the gelatin hydrogel concentration was found to significantly help in decreasing the weight loss, which indicates the possibility to control the degradation rate by changing the hydrogel formulation as reported by Zhao et al. (2016) and coworkers. Also, the addition of GNP as a crosslinker was able to decrease dramatically the degradation rate in the scaffolds. The same results were reported by Mh Busra et al. (2019); Ke et al.; Ren et al. (2017). For skin tissue engineering, it follows that the scaffold degradation rate coincides with the generation rate of new skin (Nam et al., 2012); (Branco da Cunha et al., 2014).

#### 3.1.4 Contact angle

The water contact angle (Figure 2G) is important for determining hydrogel hydrophilicity or hydrophobicity, an essential factor in drug delivery, cell proliferation and adherence. The contact angle results of all groups are shown in Figure 2C. The contact angle revealed a range from (12.1° ± 1.2°) to (9.5° ± 0.9°). No significance association was found among the groups. This shows that our scaffolds have very hydrophilic properties.

#### 3.1.5 Compression and resilience

The compression and resilience results of all groups are shown in Figures 2D, E, respectively. The NC_GEL5% showed the highest value in compression, without a significant difference, compared to other groups with (37.5% ± 4.7%). The lowest value was recorded in CL_GEL10% (7.03% ± 0.2%) followed by CL_GEL5%, and CL_GEL8% with (9.1% ± 1.7%) and (12.6% ± 1.3%), respectively. The NC_GEL10% recorded the highest resilience value with (167.3% ± 8.9%) followed by CL_GEL3% (143.7% ± 3.5%) and CL_GEL5% (135.9% ± 1.4%). The lowest resilience value was in CL_GEL8% (101.4% ± 0.2%) followed by NC_GEL8% (113% ± 0.6%). All groups represent a high resilience value superior than 100%.

The appropriate mechanical stability and the ability of the scaffolds to resemble the skin stiffness are crucial to select the appropriate biomaterial (Nam et al., 2012), (Xie et al., 2017). In the current study, the compression tests showed compression values between 7% and 37% in CL_GEL5%, CL_GEL8%, and CL_GEL10%, which suggest that gelatin concentration plays a crucial role in improving mechanical strength. It has been reported that fibroblasts show thick stress fibers when cultured on rigid substrate compared to soft scaffolds where the stress fibers are less thick or even absent (Nam et al., 2012), (Xie et al., 2017). All groups had resilience rates more than 100. High resilience shows optimum hydrogel elasticity, which is desired for shape recovery during application to maintain its efficacity (Liu et al., 2016). Resilience and adhesive force properties could be improved with GNP crosslinking by creating an intermolecular bridge between gelatin molecules through the covalent bond (Kirchmajer et al., 2013), (Arif et al., 2020). The crucial contribution of gelatin hydrogels towards strengthening mechanical properties has also been demonstrated in previous studies (Rodríguez-Rodríguez et al., 2019), (Arif et al., 2020).

#### 3.1.6 Degree of crosslinking

The degree of crosslinking results of all groups are shown in Figure 2F. The results show that the CL_GEL3% has the highest degree of crosslinking followed by the CL_GEL8% with (52.49% ± 8.8%) and (52.02% ± 2.5%), respectively. The poorest degree of crosslinking was recorded in the CL_GEL10% followed by CL_GEL5% with (26.76% ± 2.7%) and (30.72% ± 3.4%), respectively.

#### 3.1.7 Porosity and scanning electron microscope

**FIGURE 3:**
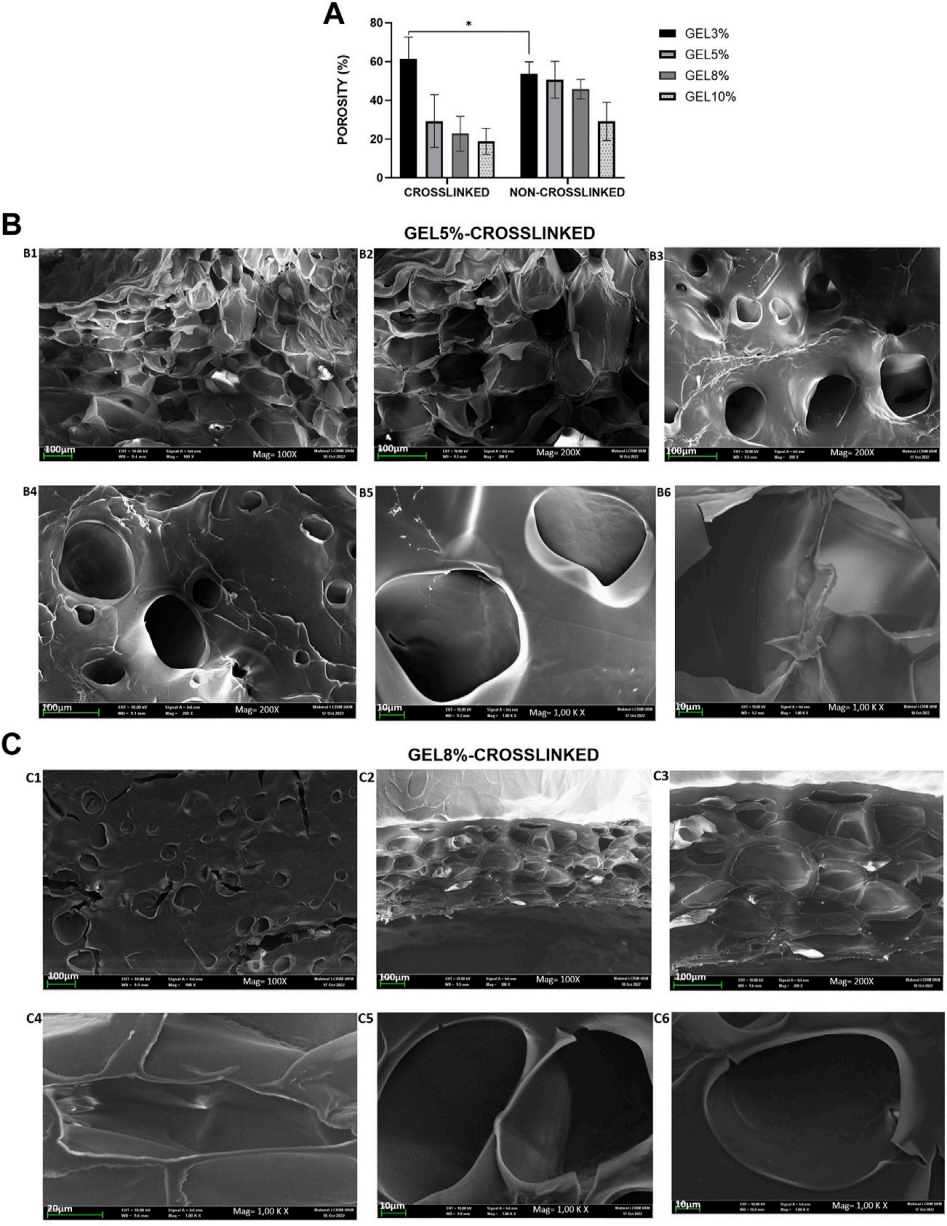
**(A)**: Physical evaluation: porosity of gelatin hydrogel scaffolds non-crosslinked and crosslinked, i.e., GEL3%, GEL5%, GEL 8%, and GEL10%. Overall, the non-crosslinked groups demonstrated higher porosity properties compared to crosslinked groups. **(B)** View under scanning electron microscope of **(B)** CL_Gel5% at magnification of ×100, scale 100 µm (B1); magnification of ×200, scale 100 µm (B2–B4); and magnification of 1.00 K X, scale 10 µm (B5-B6). **(C)** View under scanning electron microscope of CL_Gel8% at magnification of ×100, scale 100 µm (C1-C2); magnification of ×200, scale 100 µm (C3); magnification of 1.00 KX, scale 20 µm (C4) and); magnification of 1.00KX, scale 10 µm (C5-C6).

The porosity, which is the percentage of void volume in a material, was determined as shown in Figure 3A. The CL_GEL 3% hydrogel scaffold demonstrated the highest porosity property (66.88% ± 5.76%) compared to the other experimental groups followed by the NC_GEL 3% (53.81% ± 2.03%) and NC_GEL 5% (50.68% ± 3.18%). Meanwhile, the CL_GEL 10% (18.87% ± 2.22%) had the poorest porosity property, followed by the CL_GEL 8% (27.84% ± 5.54%).

The results of the scanning electron microscope (SEM) of CL_GEL5% and CL_GEL8% are shown in Figures 3B, C, respectively. SEM analysis showed that the CL_GEL 5% scaffold had interconnected pores with size range of 40–183 μm. The SEM analysis showed that the CL_GEL8% scaffold had interconnected pores with size range of 39–170 μm.

The biomaterial properties of the scaffolds are vital in determining the cells behavior and the full reepithelization of the tissue. Therefore, the 3D scaffolds should be highly porous with interconnected pores to enable the diffusion of oxygen, nutrient and the waste removal (Loh and Choong, 2013). According to earlier research, porous materials with a range within 20–125 µm are optimum to reconstruct adult skin (Sisso et al., 2020); (Rodríguez-Rodríguez et al., 2020); (El-Sherbiny and Yacoub, 2013) and pore sizes within 40–600 µm promote capillary ingrowth (Crowley et al., 2016); (de Mel et al., 2009). The SEM results of the CL_GEL5% and the CL_GEL8% show that the range of the pore sizes is within 40–183 μm and 39–170 μm; respectively, which is acceptable for tissue regeneration. However, The porosity of all four hydrogel formulations was between 18% and 60% this is fell short of several other biomaterials studied in the literature, which might approach 80%–90% porosity (Wang et al., 2016), (Mahboudi et al., 2015). The porosity and pore size determine the ultimate mechanical quality of the scaffold, which affects cell behavior (Mahboudi et al., 2015).

#### 3.1.8 Energy dispersive X-Ray (EDX) composition analysis

The results of the energy dispersive X-Ray composition analysis of CL_GEL5% and CL_GEL8% groups are shown in Supplementary Figure S1A. Elemental study of CL_GEL5% and CL_GEL8% treatment groups revealed three main components, including nitrogen (N; 11%), carbon (C; 60%–80%), and oxygen (O; 25%–30%) in CL_GEL5%, and (C; 55%–60%), (N; 25%),(O; 10%–25%) in CL_GEL8%, as shown in Supplementary Figures S1A1, A2, respectively.

#### 3.1.9 Fourier transform infrared spectrometry

The Fourier transform infrared spectrometry (FTIR) results of CL_GEL5% and CL_GEL8% are presented in Supplementary Figures S1B1, B2, respectively. The FTIR revealed 4 regions, the region A, B, C and D. The IR spectra of CL_GEL8% and CL_GEL5% showed similar absorbances resembling the Amide A (3,400–2,300 cm^−1^), Amide I (1,600–1,650 cm^−1^), Amide II (1,530–1,200 cm^−1^), and Amide III (1,230–670 cm^−1^). No major shift was prominent in FTIR spectra in both formulations after genipin modification as described in a previous study (Nike et al., 2021). Briefly, at the A region, the amine group attack O-H group. CL-GEL8% had highest O-H group (3,285.94 cm^−1^) and lower crosslinking followed by CL_GEL5% (3,283.59 cm^−1^). The region B and C showed that CL_GEL5% had the highest Amide I (1,631 cm^−1^) and Amide II (1,544.78 cm^−1^) followed by CL_GEL8% (Amide I (1,629.55 cm^−1)^) (Amide II (1,541.58 cm^−1^). At region D, both CL_GEL5% and CL_GEL8% presented almost the same level of Amide III (1,238.32 cm^−1^ and 1,238.72 cm^−1^), respectively**.**

#### 3.1.10 X-ray diffraction

The X-ray diffractogram of fabricated hydrogels demonstrated almost similar patterns for both CL_GEL5% and CL_GEL8% treatment groups as shown in Supplementary Figures S1C1, C2, respectively. All diffractograms represented a broad peak at 2θ in between 10° to 50°. The XRD patterns for CL_GEL5% described CL_GEL8% due to similar gelatin initial stock except for its concentration. CL_GEL5% presented the highest percentage of crystallinity with 29.3% and 70.7% of amorphous followed by CL_GEL8% with 15.5% of crystallinity and 84.5% of amorphous. As expected, the EDX, FTIR and the XRD analysis demonstrated that GNP did not affect the native chemical properties of gelatin.

### 3.2 Biocompatibility characterization

#### 3.2.1 Cell isolation and culture (keratinocytes and fibroblasts)

**FIGURE 4:**
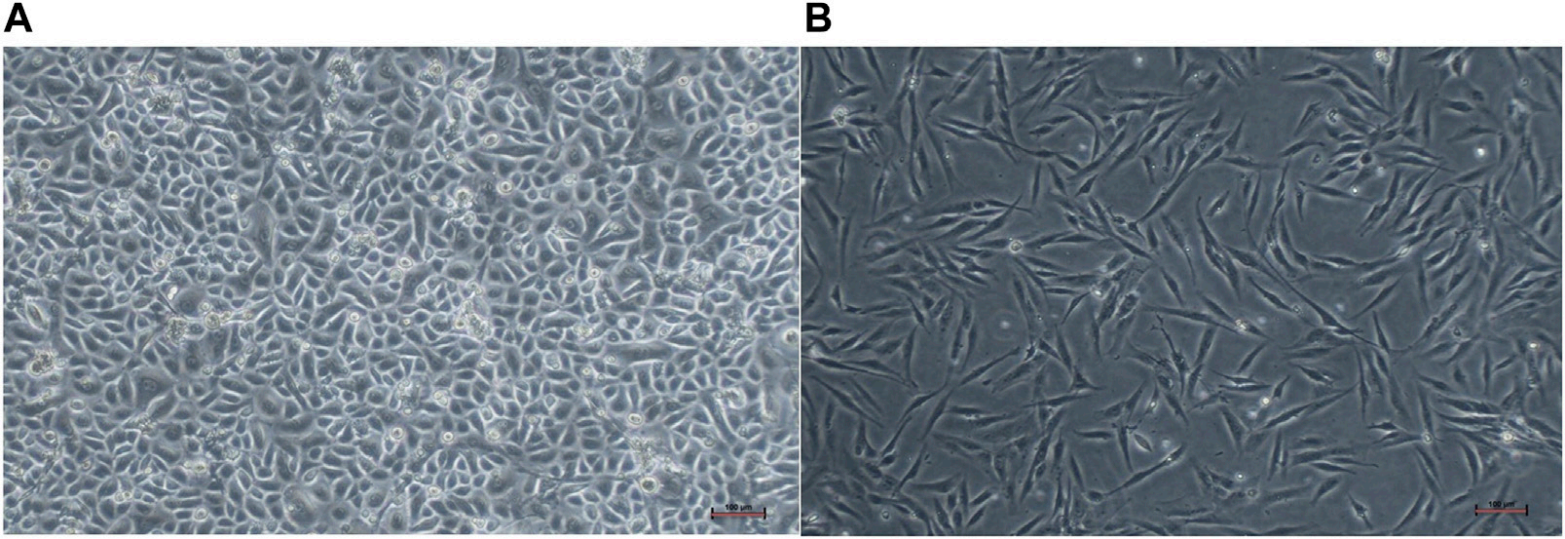
Monolayer cells cultured in 6-well plate: **(A)** culture of human epidermal keratinocytes (HEKs), and **(B)** human dermal fibroblasts (HDFs), The cultured cells became confluent in a week’s time and fibroblasts were separated by differential trypsinization before seeding into a new flask. Scale bar: 100 μm, magnification ×4.

The primary culture of human epidermal keratinocytes and human dermal fibroblast were successfully established from skin samples (Figure 4).

#### 3.2.2 Cell attachment

**FIGURE 5:**
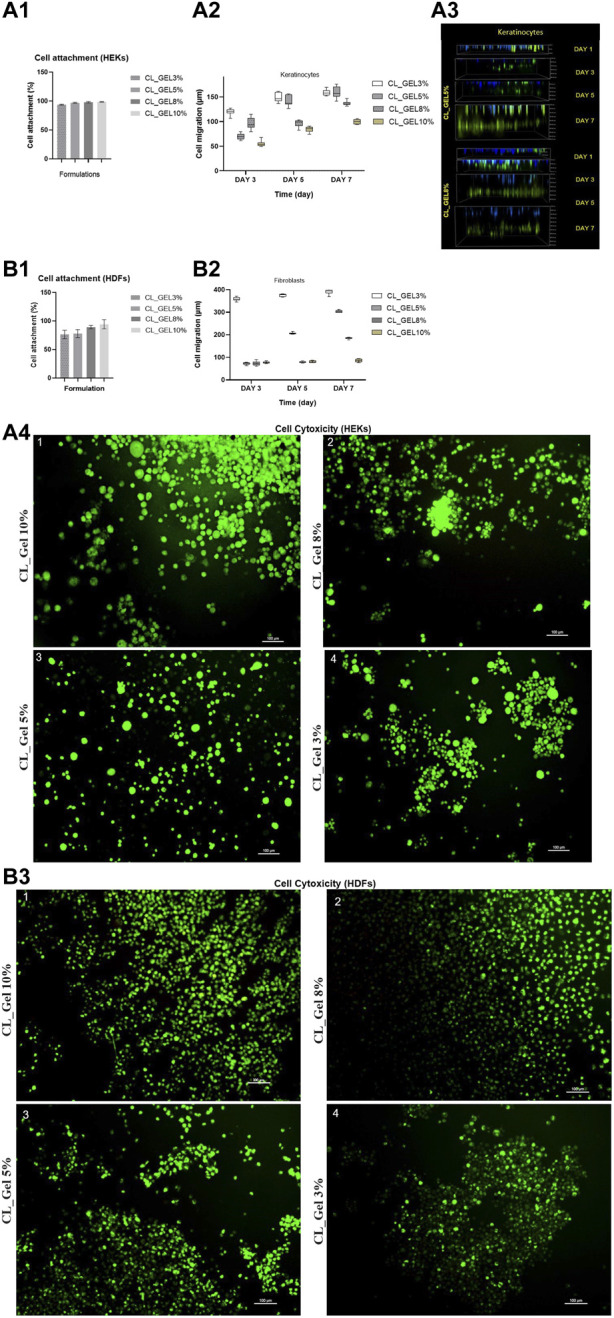
Cell attachment (%) of **(**A1): human epidermal keratinocytes (HEKs) and (B1): human dermal fibroblasts (HDFs) in CL_GEL3%, CL_GEL5%, CL_GEL8%, and CL_GEL10% formulations after 24 h. Cell migration (µm) of (A2) keratinocytes and (B2) fibroblasts of CL_GEL3%, CL_GEL5%, CL_GEL8%, and CL_GEL10% at various interval times (day 3, 5 and 7). (A3) Three-dimensional (3D) confocal image of cell migration (µm) on days 1, 3, 5 and 7 of CL_GEL5% and CL_GEL8% of keratinocytes (Green: cell tracker; Blue: Hoescht dye), the green color demonstrated that the cells are migrating from an upper chamber to a lower chamber through a microporous membrane. Live/dead staining of (A4) human epidermal keratinocytes (HEKs) and (B3) human dermal fibroblasts (HDFs) seeded on (1) CL_GEL10%, CL_GEL8%, CL_GEL5% and CL_GEL3%, after 24 h. The results revealed non-cytotoxic effect of gelatin hydrogel crosslinked with genipin. The green color demonstrated live (HEKs) and (HDFs) cells. Scale bar = 100 µm (10X). (Green: live cells; Red: dead cells).

HDFs and HEKs were seeded on top of the gelatin hydrogel scaffolds (CL_GEL3%, CL_GEL5%, CL_GEL8%, and CL_GEL10%), and the efficiency of cell attachment was evaluated at 24 h after seeding (Figures 5A1, B1). All groups showed a high attachment property and the CL_GEL 10% (98.75% ± 0.38%) scaffolds demonstrated the highest attachment of HEKs compared to other scaffolds followed by the CL_GEL 8% (98.16% ± 0.72%), CL_GEL 5% (97% ± 0.57%) and CL_GEL 3% (93.83% ± 0.44%). The same observation goes with the HDFs where the CL_GEL 10% showed the highest attachment property with (94.33% ± 4.66%) followed by CL_GEL 8% (89.33% ± 1.7%), CL_GEL 5% (78% ± 4%) and CL_GEL 3% (76.33% ± 4.33%). The attachment properties of the gelatin hydrogel scaffolds increased proportionally with the concentration of gelatin. Additionally, the scaffolds with different formulations presented a higher attachment property for HEKs than HDFs.

Our fabricated 3D gelatin hydrogel showed favorable characteristics in terms of stability and in supporting the attachment of HDFs and HEKs as expected. The properties that may affect cell attachment like hydrophilicity, pore size, surface roughness, were almost similar between the different formulations and were favorable for cell attachment. This suggests that the gelatin hydrogel exhibits rapid cell attachment as more than 90% of HEKs and HDFs are already attached after 24 h. The excellent attachment rate may be explained by the hydrophilicity of the hydrogel and the surface area reasonably available for the cells to attach as the porosity is rather low. This property is very important for cell function including cell spreading, migration and proliferation (Anselme et al., 2010).

#### 3.2.3 Cell toxicity assessment

HEKs and HDFs were seeded on top of the gelatin hydrogel scaffolds, and the efficiency of cell attachment and viability were evaluated at 24 h after seeding as shown in Figures 5A4, B3, respectively. All groups showed a high attachment property and high viability. The CL_GEL 10% scaffolds demonstrated the higher attachment and viability of HDFs compared to other scaffolds. All scaffolds demonstrated a high attachment and viability of HEK.

The results for the cell viability assay demonstrated a high number of live cells and a very low number of dead cells on all formulations of hydrogel, this confirms that the hydrogel supports cell viability and provides nontoxic 3D scaffolds.

#### 3.2.4 Cell migration

The cell migration results of HEKs and HDFs are shown in Figures 5A2, B2, respectively. Figure 5A3 showed HEKs migrating from an upper chamber to a lower chamber (upper chamber cells stained in blue, migrated cells stained in green). HEKs and HDFs did migrate into the hydrogel as the cells did not and remained at the top surface on day 3, 5 and 7 unlike the first day where the cells seemed to be at the same level with the attached cells in the plate (upper chamber).

At the third day, the highest distance of migration in HEKs (µm) was recorded in CL_GEL3% followed by CL_GEL8% with (119.8 ± 2.01 µm) and (96.23 ± 3.8 µm). The lowest distance of migration was recorded in CL_GEL10% with (55.2 ± 1.7 µm). For HDFs, the CL_GEL3% showed the highest distance of migration with (359.29 ± 2.5 µm) followed by CL_GEL10% with (76.8 ± 1.4 µm) and the lowest migration distance for HDFs was recorded in CL_GEL5% with (72.3 ± 1.5 µm), but no significant difference was observed between CL_GEL5% and CL_GEL8%. The results showed a significant difference between CL_GEL3% and all other groups at the third day.

At the fifth day, HEKs showed the highest distance of migration in the hydrogel CL_GEL3% followed by the CL_GEL5% with (149.96 ± 3.1 µm) and (142 ± 3.7 µm), respectively. The lowest distance was recorded also in CL_GEL10% with (84.22 ± 1.8 µm). The results showed a significant difference among all the groups except for CL_GEL3% and CL_GEL5%. HDFs showed the highest distance of migration in the hydrogel CL_GEL3% followed by the CL_GEL5% with (374.66 ± 1.9 µm) and (206.36 ± 1.2 µm), respectively. The lowest distance was recorded in CL_GEL8% and CL_GEL10% with (79.02 ± 1.2 µm) and (80.58 ± 1.5 µm), respectively with no significant difference. The results show a significant difference among the other groups.

At the seventh day, the HEKs migrated the longest distance in CL_GEL5% followed by CL_GEL3% with (160.01 ± 3.9 µm) and (158.56 ± 2 µm), respectively with no significant difference, while the shortest distance was reported in CL_GEL10% with (99.74 ± 1.5 µm). All others groups showed significant difference. HDFs showed the highest distance of migration in the hydrogel CL_GEL3% followed by the CL_GEL5% and the CL_GEL8% with (390.75 ± 3.5 µm), (303.36 ± 1.6 µm), and (184.8 ± 1.2 µm), respectively. The lowest distance was recorded in CL_GEL10% with (84.34 ± 2.2 µm). The results showed significant difference among all groups.

Cell migration within scaffolds is crucial for skin tissue adaptation. The results of cell migration on different formulations were significantly different among groups. The CL_GEL3%, CL_GEL5%, and CL_GEL8%, showed positive results in cell migration after 7 days with a range of 390 μm, 303 μm and 184 µm in HDF and 158 μm, 160 μm, and 136 µm in HEK; respectively. It has been reported that the optimum pore size for fibroblasts is between 20 and 120 µm which goes along with our results in CL_GEL5% and CL_GEL8%. This suggests that the pore sizes within 39–170 and the pore size within 40–183 µm are suitable for HEKs and HDFs migration on gelatin hydrogel. The scaffolds should promote HEKs and HDFs adhesion and migration and allow the retention of metabolic functions of attached cells. However, The CL_GEL10% did not allowed the migration of HDFs and HEKs which may be due to the small pore sizes caused by the increasing of the gelatin concentration. This indicates that the CL_GEL10% is not suitable for cell migration. Oliveira Barud et al. (2015), reported that even with an average pore size of 102 ± 5.43, no noticeable migration of fibroblasts was observed on bacterial cellulose/50% fibroin scaffold and they suggest that this is due to the dense network that do not generate enough pore size to facilitate cell migration. We suggest also that the CL_GEL10% did not promote the HEKs and the HDFs, perhaps, because of the dense network created by gelatin-genipin hydrogel that does not possess enough pores with the suitable size for the cell migration compared to all other formulations. It is noteworthy that the HDFs migrate better than HEKs which could be due to their capacity to adapt.

#### 3.2.5 Immunocytochemistry

**FIGURE 6:**
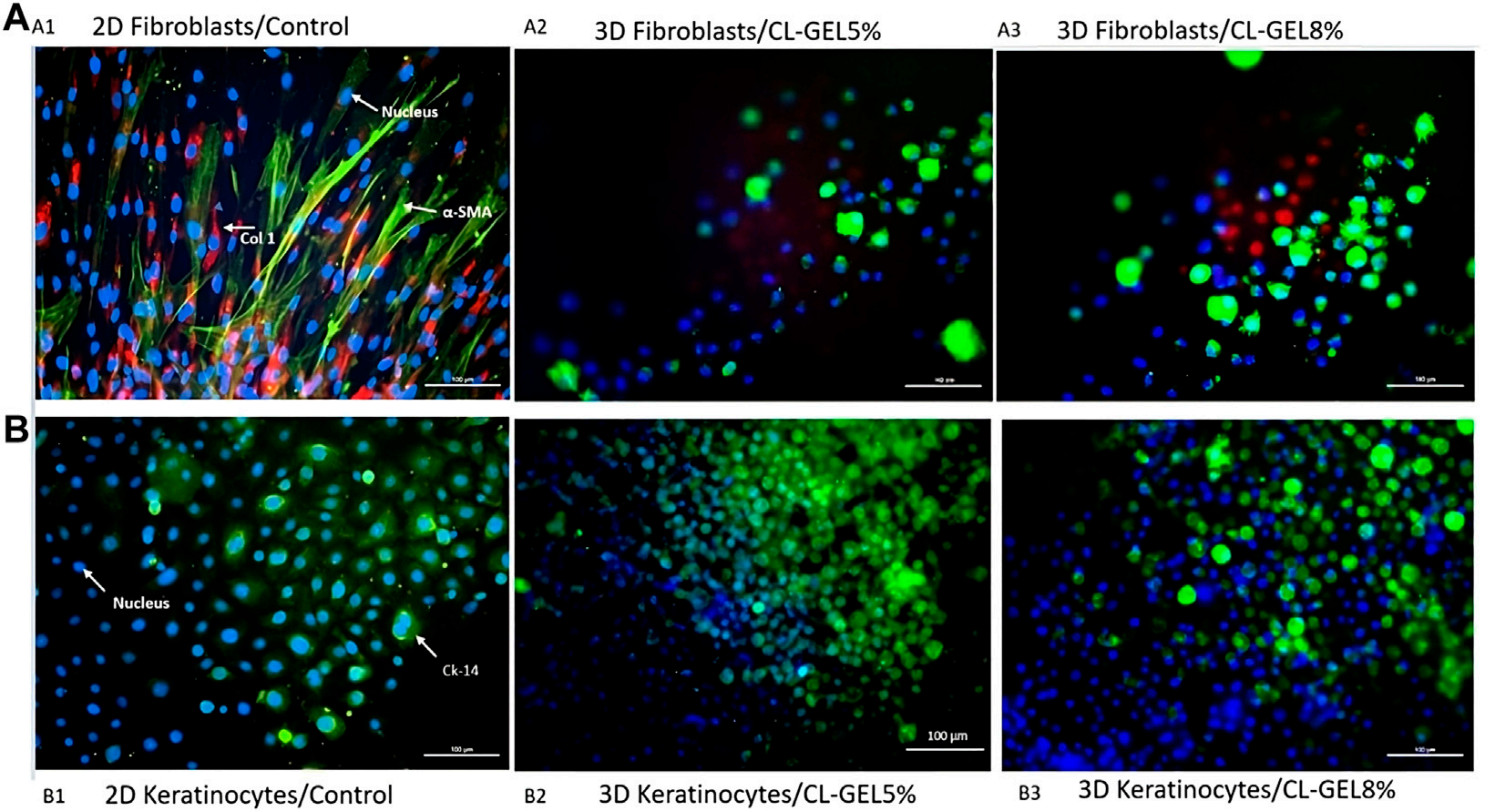
Immunocytochemistry staining of collagen type 1, α-SMA, and cytokeratin 14. **(A)** Immunocytochemistry staining of human dermal fibroblasts (HDFs) on (A1) monolayer 2D culture as control; (A2) CL_GEL5% and (A3) CL_GEL8%. The cells were stained with immunofluorescence (Alexa 594, red; COL I), (Alexa 488, green; α-SMA) and with Hoechst (blue; cell nuclei). HDFs on both CL_GEL5% and CL_GEL8% demonstrated the presence of α-SMA (green) positive cells; and collagen type 1 (red) positive cells. **(B)** Immunocytochemistry staining of human epidermal keratinocytes (HEKs) on (B1) monolayer 2D culture as control; (B2) CL_GEL5% and (B3) CL_GEL8%. The cells were stained by immunofluorescence (Alexa 488, green; CK-14) and with Hoechst (blue; cell nuclei). HEKs on both CL_GEL5% and CL_GEL8% demonstrated the presence of cytokeratin 14 (green) positive cells. Scale bar: 100 µm.

HDF on both CL_GEL5% and CL_GEL8% revealed the positive expression of α-SMA (green); and collagen type 1 (red) as shown in Figures 6A2, A3, respectively. However, the number of α-SMA and collagen type 1 positive cells was low compared to that of total cells. In addition, if we compare the cell behavior on the hydrogel and on the 2-dimentional (2-D) cell culture control (Figure 6A1), the number of the adherent cells was significantly higher in the control in both groups. On the other hand, HEK on both scaffolds showed the presence of cytokeratin 14 (green) and the number of cytokeratin 14 positive cells was high (Figures 6B2, B3). Interestingly, the number of the adherent cells in the CL_GEL5% and the CL_GEL8% was higher than the 2D control (Figure 6B1).

In order to investigate the maturation process, the changes in the expression of cytokeratin 14 in HEKs and the expression of α-SMA and collagen type 1 in HDFs were examined. The cytokeratin 14, a type 1 keratin, is mainly located in the basal keratinocytes of the epidermis, stratified epithelia and in the hair follicle’s exterior root sheet (Seet et al., 2012). The positive results suggest that CL_GEL5% and CL_GEL8% promote the differentiation of keratinocytes and fibroblasts. The increased expression of this marker of basal keratinocytes proliferating in the CL_GEL5% and CL_GEL8% groups compared to the 2-D control indicated more layers of proliferating keratinocytes, which suggests that HEKs were in an earlier phase of maturation. On the other hand, collagen type 1 was highly expressed in 2-D fibroblasts culture, which suggests that there was no disruption of collagen type 1 level. However, collagen type 1 was scarcely present in the CL_GEL5% and CL_GEL8% groups, which suggests that the HDFs are still in the earlier phase of maturation compared to 2-D controls. The results showed that there was more collagen type 1 in the control compared to the hydrogel groups loaded with cells which does not match the cell viability and cell attachment findings. It is known that during the remodeling and maturation phase of wound healing, collagen type III is remodeled to collagen type 1 (Miedel et al., 2015), which supports the finding that the treatment using hydrogel loaded with cells promote regeneration faster than using the hydrogel alone. However, this our results contradict this observation as the hydrogel does not promote the HDFs maturation.

In addition, The expression of α-SMA is linked to myofibroblasts, which are involved in tissue contraction (Desmouliere et al., 2014). In physiological remodeling, the contractile activity of myofibroblasts is terminated when the tissue is repaired; α-SMA expression subsequently decreases and myofibroblasts are removed by apoptosis (Desmoulière et al., 1995). In our experiments, the expression of α-SMA in the 2D control group was the highest, which suggests that differentiation is much faster compared to the gelatin hydrogel groups.

#### 3.2.6 Cell proliferation

**FIGURE 7:**
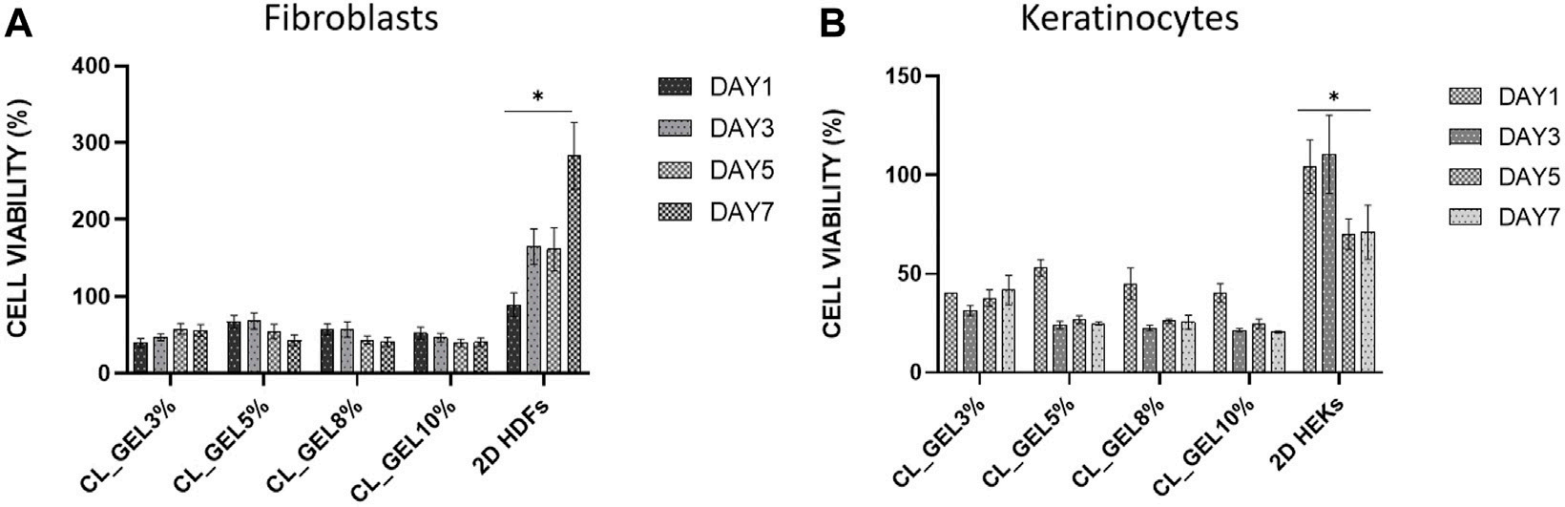
Cell proliferation of **(A)** human dermal fibroblasts (HDFs) and **(B)** human epidermal keratinocytes (HEKs) seeded on gelatin hydrogel 3D scaffolds (CL_GEL3%, CL_GEL5%, CL_GEL8%, and CL_GEL10%) and 2D controls after 1,3,5 and 7 days. HDFs and HEKs proliferations were too low compared to the 2D culture controls. *Control group significantly higher compared to other groups for both cell types.

MTT assay confirmed that HDFs and HEKs proliferations were too low compared to the 2D culture control as shown in Figures 7A, B, respectively. At the third day, the CL_GEL5% showed the highest cell viability in HDFs followed by CL_GEL8% with 68.033 ± 5.3 and 53.03 ± 4.9; respectively compared to 164.77 ± 11.5 in 2D HDFs control. This rate decreased to 42.86 ± 3.55 and 40.84 ± 3 in CL_GEL5% and CL_GEL8%; respectively after 7 days. However, the cell viability in control increased to 282.88 ± 21.8. The cell viability in HEKs was lower compared to HDFs. At the third day, the highest cell viability was recorded in CL_GEL3% followed by CL_GEL5% and CL_GEL8% with 31.31 ± 1.5, 23.95 ± 1.1 and 22.6 ± 0.7; respectively. The cell viability was higher in 2D HEKs control with 110.4 ± 11.4. After 7 days, the cells proliferate slightly in CL_GEL3% with 41.67 ± 4.3, in CL_GEL8% with 25.5 ± 2.03 and in CL_GEL5% with 24.77 ± 0.45 which is lower compared to the 2D HEKs control (71.05 ± 7.9). All groups showed low viability and proliferation of HDFs and HEKs compared to 2D controls.

Cellular compatibility is another concern for an ideal bioscaffold for skin tissue engineering to maintain viability and support human skin cells proliferation. Unfortunately, all gelatin hydrogel groups showed a negative proliferative effect on HDFs and HEKs as the proliferation was either too low or decreasing which is not consistent with previous findings from Ilkar Erdagi et al. (2020). Unfortunately, the proliferation of HDF at the top surface of hydrogels was decreased after 7 days incubation and the proliferation of HEKs was slightly increased but still very low compared to the control. This phenomenon is probably related to its mechanical strength, as was reported previously by Lee et al. (2014) who concluded that a limited proliferative effect occurred in the hydrogel with higher stiffness due to slower degradation and lower permeability (Gupta et al., 2019).

#### 3.2.7 Histological and immunohistochemical analysis of skin maturation

**FIGURE 8:**
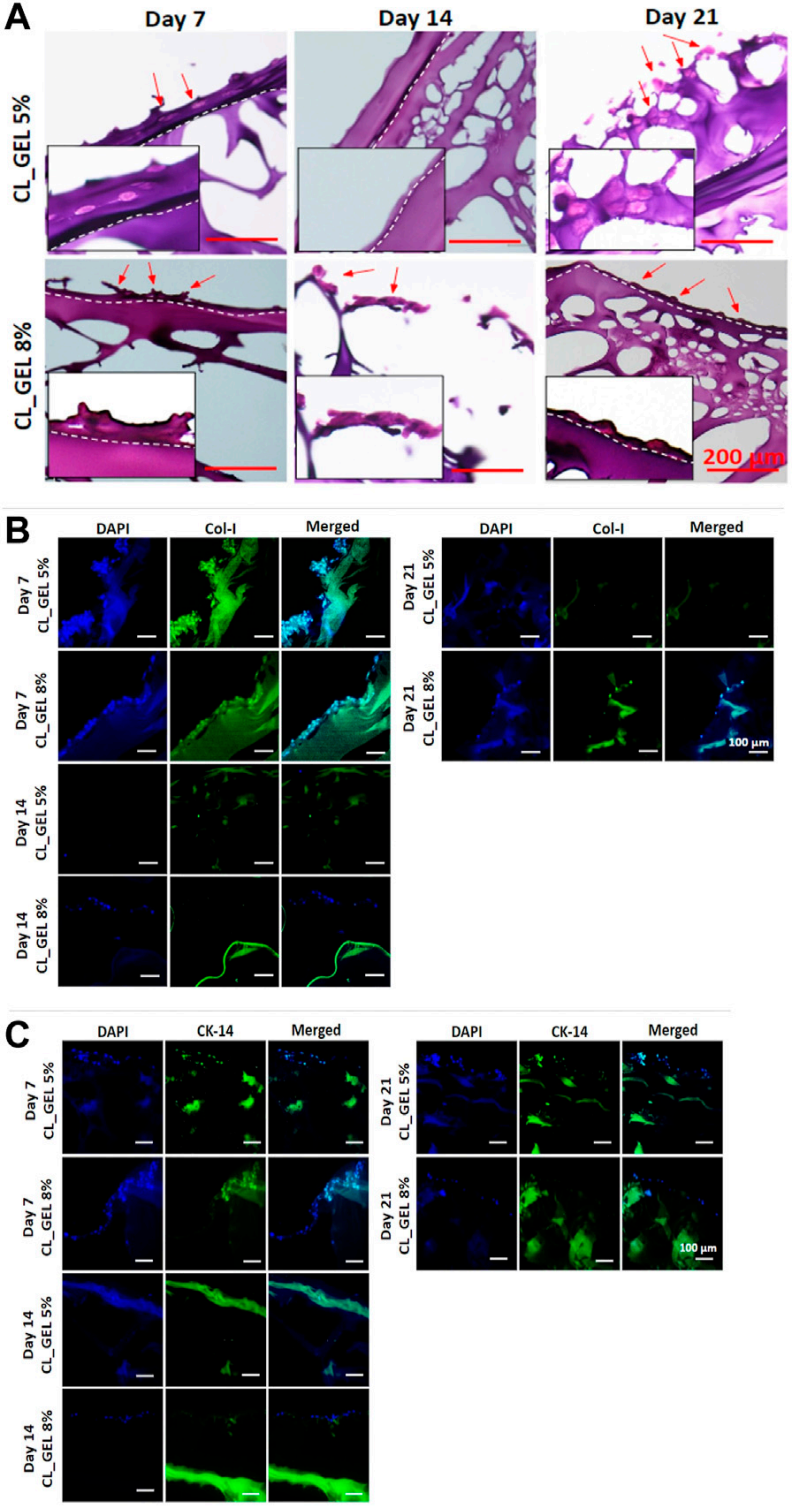
Microscopic evaluation of hydrogel model **(A)** H&E-stained images of hydrogels performed on day 7, 14, and 21 (scale bar: 200 μm), **(B)** immunofluorescent images of hydrogels to detect the presence of collagen type 1 on day 7, 14, and 21 (×20 magnification), **(C)** immunofluorescent images of hydrogels to detect the presence of cytokeratin 14 on day 7, 14, and 21 (×20 magnification).

The H&E staining was carried out to verify the maturity level of the 3D *in vitro* skin model using hydrogels. For comparison, we used both HEKs and HDFs cells to construct *in vitro* skin models using hydrogels. Figure 8A displays the H&E-stained images of the hydrogels under different incubation periods (7, 14, and 21 days). From day 7 until day 21 incubation, the presence of stratified keratinocytes that are exposed to air was indicated in both CL_GEL 5%, and CL_GEL 8%. It can be seen from the images that about two layers occurred which indicates the separation of epidermis and dermis layer. Nonetheless, due to an insufficient incubation period, both formulations were unable to attain sufficient maturity to form a suprabasal layer or thicken the spinous layer. In addition, there is no presence of fibroblasts cells in the inner layer of the hydrogels.

#### 3.2.8 Immunofluorescent analysis of skin maturation

The skin model was constructed by cultivating mixed skin cells (HEKs and HDFs) onto gelatin hydrogels. Thus, we examined the expression marker of collagen type 1, and cytokeratin 14 using immunofluorescent staining. Figure 8B showed the immunofluorescent stained images of the hydrogels using collagen type 1 marker for day 7, 14, and 21. Results showed that collagen type 1 staining was present in both CL_GEL 5% and CL_GEL 8% microstructure Day 7 only. This indicates that there is no HDFs in both CL_GEL 5% and CL_GEL 8% on day 14 and 21 incubation period. Moreover, Figure 8C shows the IF-stained images of hydrogels using cytokeratin 14 marker for day 7, 14, and 21. Cytokeratin 14 is known as an important marker for epidermal basal cell detection. The results indicate that CL_GEL 5% and CL_GEL 8% expressed higher level of cytokeratin 14 on day 7 while CL_GEL 8% slightly expressed cytokeratin 14 on day 14. This indicates that the 3D *in vitro* models are not fully matured after 21 days of incubation.

Complex biofabricated scaffolds cannot be employed immediately after fabrication as *in vitro* tissue models. The generation of a fully functional skin equivalent that closely resembles the structure and functionality of native skin is required for the maturation of a hydrogel-based 3D *in vitro* skin model. This includes the successfully differentiation of keratinocytes to form epidermis layers, and the development of dermal compartment with fibroblasts and extracellular matrix.

Generally, tissue maturation requires ideal circumstances, including culture environment, time, and medium composition (nutrition) to support cell growth. Based on our findings, the presence of separation layers between epidermis and dermis. This phenomenon have been occurred and explained by Kwak et al. (2018) who mentioned that the epidermis’ differentiation was triggered by air exposure. Moreover, our histological findings also found that the 3D *in vitro* model using CL_GEL 5%, and CL_GEL 8% were not fully matured after 21 days incubation period. Longer cultured of HEKs and HDFs in the hydrogels did not increase the expression of collagen type 1 and cytokeratin 14. This finding was similar with a previous study by Kim et al. (2019) who developed a 3D *in vitro* model using hydrogels claimed that it is not sufficient to prove that their skin model recapitulates a more accurate and predictive *in vivo* response given in the milieu of original skin.

## 4 Conclusion

Our three-dimensional (3D) scaffolds demonstrated good physical and chemical properties (hydrophilic, crosslinked polymeric networks, high swelling rate, biodegradation) while maintaining their 3D structure except for Gel3% that was excluded. All gelatin hydrogel groups were compatible for skin tissue engineering applications in cellular attachment, cell viability but not for cell proliferation. Considering that the intended use of the hydrogel is to serve as a skin substitute, the ability to maintain cell attachment and viability is not sufficient for its function as cell proliferation deemed important to ensure a full regeneration of the HDFs and HEKs. Insufficient incubation time and the absence of additional measures to enhance cell differentiation could represent limitations in our study. We require a longer period of incubation and additional adjustments to further stimulate cell differentiation and regeneration. Future studies could focus on modifying the hydrogel composition to enhance its ability to promote cell proliferation and further investigate the potential of these 3D scaffolds for skin tissue engineering applications. We also suggest exploring the use of different cell concentrations and increasing the initial number of cells to address the limitations of skin maturation in our model.

The 3D *in vitro* skin model made from gelatin hydrogel has several applications, including as an alternative to animal experiments in preclinical studies, for screening for toxicity of various substances, for studying wound healing and disease modeling, and for developing personalized medicine. Overall, this model has the potential to provide a more accurate and reliable testing platform, accelerate the development of new treatments for skin-related conditions, and reduce the need for animal testing.

## Data Availability

The original contributions presented in the study are included in the article/Supplementary Material, further inquiries can be directed to the corresponding author.

## Supplementary material

SUPPLEMENTARY FIGURE S1 Chemical characterization. **(A)** Energy dispersive X-Ray (EDX) map and spectra gave information that the presence of the element in the surface of (A1) CL_GEL5% and (A2) CL_GEL8% were similar to NC_GEL5% and NC_GEL8% (data not shown). **(B)** Fourier Transform Infrared Spectrometry (FTIR) spectra of (B1) CL_GEL5% and (b2) CL_GEL8%. **(C)** X-ray diffractogram of (C1) CL_GEL5% and (C2) CL_GEL8% confirmed that genipin modification did not alter the origin functional groups and amorphous nature of gelatin.
