# Supplementary figures and images for: Human epidermal keratinocytes and human dermal fibroblasts interactions seeded on gelatin hydrogel for future application in skin in vitro 3-dimensional model
Source: Front Bioeng Biotechnol. 2023 Jun 23;11:1200618. doi: 10.3389/fbioe.2023.1200618 (PMC10326847; doi:10.3389/fbioe.2023.1200618)

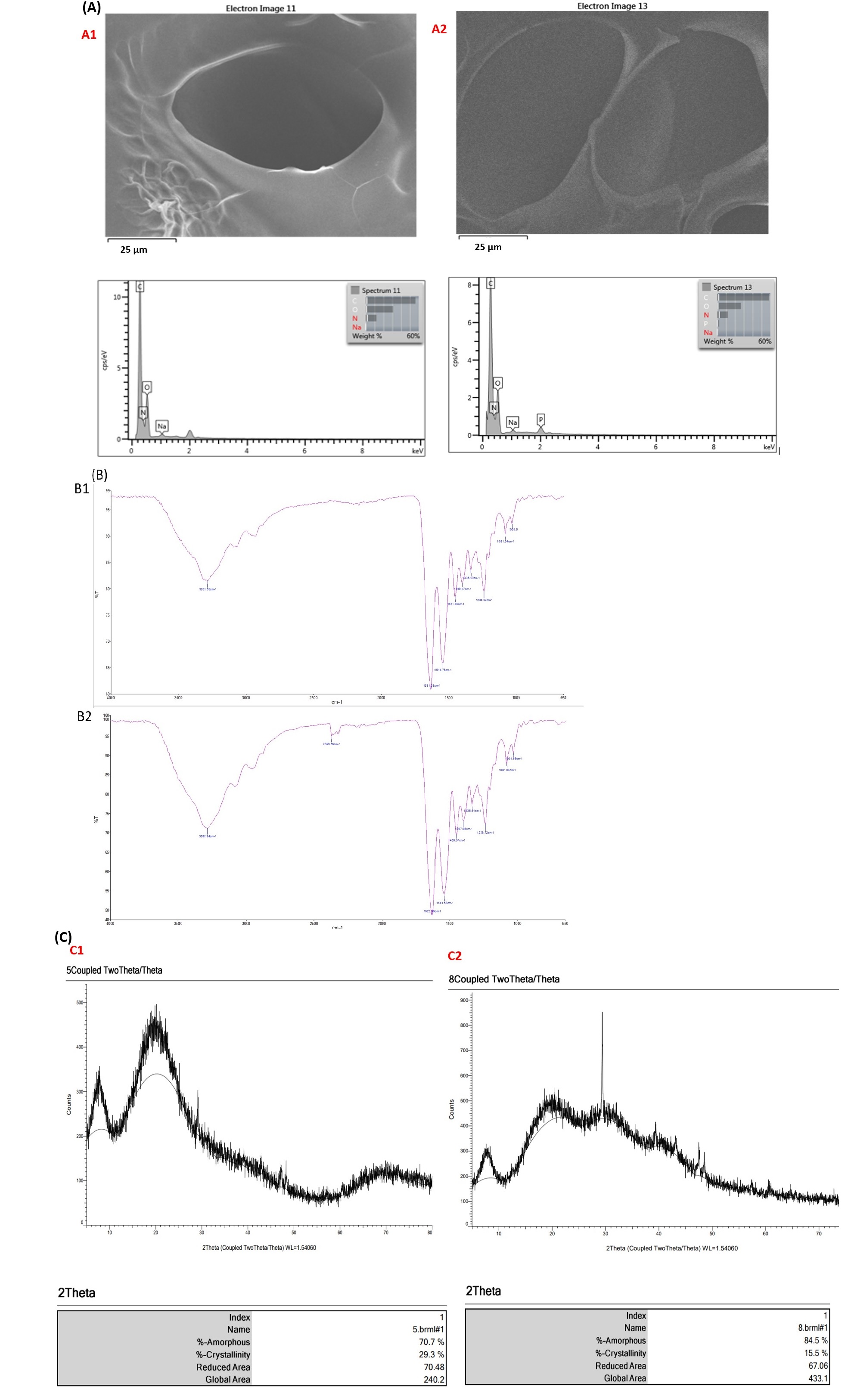

Supplement: Supplementary file 1 [file Image1.JPEG]
